# Locomotor behavior and hearing sensitivity in an early lagomorph reconstructed from the bony labyrinth

**DOI:** 10.1002/ece3.9890

**Published:** 2023-03-18

**Authors:** Sergi López‐Torres, Raj Bhagat, Ornella C. Bertrand, Mary T. Silcox, Łucja Fostowicz‐Frelik

**Affiliations:** ^1^ Biological and Chemical Research Centre, Institute of Evolutionary Biology, Faculty of Biology University of Warsaw Warsaw Poland; ^2^ Division of Paleontology American Museum of Natural History New York New York USA; ^3^ New York Consortium in Evolutionary Primatology New York New York USA; ^4^ Department of Anthropology University of Toronto Scarborough Toronto Ontario Canada; ^5^ Institut Català de Paleontologia Miquel Crusafont Universitat Autònoma de Barcelona, Edifici ICTA‐ICP Cerdanyola del Vallès Spain; ^6^ Department of Organismal Biology and Anatomy The University of Chicago Chicago Illinois USA; ^7^ Key Laboratory of Evolutionary Systematics of Vertebrates, Institute of Vertebrate Paleontology and Paleoanthropology Chinese Academy of Sciences Beijing China; ^8^ Department of Evolutionary Paleobiology, Institute of Paleobiology Polish Academy of Sciences Warsaw Poland

**Keywords:** agility, cochlea, hearing, lagomorph, locomotion, semicircular canals

## Abstract

The structure of the bony labyrinth is highly informative with respect to locomotor agility (semicircular canals [SCC]) and hearing sensitivity (cochlear and oval windows). Here, we reconstructed the agility and hearing sensitivity of the stem lagomorph *Megalagus turgidus* from the early Oligocene of the Brule Formation of Nebraska (USA). *Megalagus* has proportionally smaller SCCs with respect to its body mass compared with most extant leporids but within the modern range of variability, suggesting that it was less agile than most of its modern relatives. A level of agility for *Megalagus* within the range of modern rabbits is consistent with the evidence from postcranial elements. The hearing sensitivity for *Megalagus* is in the range of extant lagomorphs for both low‐ and high‐frequency sounds. Our data show that by the early Oligocene stem lagomorphs had already attained fundamentally rabbit‐like hearing sensitivity and locomotor behavior, even though *Megalagus* was not a particularly agile lagomorph. This is likely because *Megalagus* was more of a woodland dweller than an open‐habitat runner. The study of sensory evolution in Lagomorpha is practically unknown, and these results provide first advances in understanding the primitive stages for the order and how the earliest members of this clade perceived their environment.

## Graphical abstract

The structure of the inner ear (enclosed in the bony labyrinth) in mammals is highly informative with respect to locomotor agility and hearing sensitivity. Our data on the fossil rabbit *Megalagus* show that by the early Oligocene lagomorphs had already attained fundamentally rabbit‐like hearing sensitivity and locomotor behavior, even though *Megalagus* was not a particularly agile lagomorph.

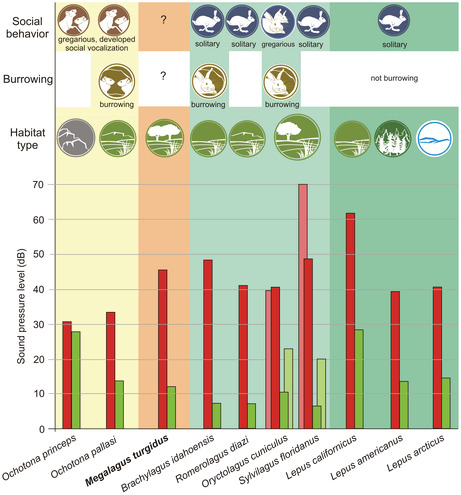

## 1 INTRODUCTION

The middle and inner ear structures in mammals are almost completely enclosed by bone and thus often very well preserved in fossils, even if the rest of the skull is poorly or not at all preserved (Meng & Fox, 1995). Specifically, the bony labyrinth (housing the inner ear) in mammals has been employed in both broad comparative and functional anatomical studies (e.g., Berlin et al., 2013; Ekdale, 2013; Gunz et al., 2012) as well as in more specialized research (see below). The ecological importance of the labyrinthine morphology stems from its potential to be informative about the animal's hearing sensitivity, sense of balance, and locomotor agility, all of which directly influence an animal's lifestyle and behavior. Numerous studies have drawn such inferences from the bony labyrinth of living and fossil primates (e.g., Bernardi & Couette, 2017; Coleman et al., 2010; Coleman & Boyer, 2012; Lebrun et al., 2010; Malinzak et al., 2012; Ryan et al., 2012; Silcox et al., 2009; Spoor et al., 2007; Walker et al., 2008); rodents (Bhagat et al., 2021; Pfaff et al., 2015); xenarthrans (Billet et al., 2015); carnivorans (Grohé et al., 2016); artiodactyls (Mennecart & Costeur, 2016); marsupials (Schmelzle et al., 2007); or extinct leptictids (Ruf et al., 2016) and “condylarths” (Bertrand et al., 2020). Whereas some studies have included modern lagomorphs into their datasets (Ekdale, 2013; Spoor et al., 2007), the lagomorph sample was not large enough to allow any understanding of this order outside the broader context of Mammalia.

Cranial material of fossil lagomorphs that predates the Oligocene is extremely rare. The only species known from a partial skull is *Dawsonolagus antiquus* from the lower part of the Arshanto Formation (late early Eocene) of Nei Mongol, China; however, the skull lacks the posteroventral part, including the ear region (Li et al., 2007). Following the first radiation of the group in the early middle Eocene of Central Asia (Fostowicz‐Frelik et al., 2015), lagomorphs quickly appeared in North America, where they have been present since the middle Eocene (ca. 42 Ma, late Uintan North American Land Mammal Age [NALMA], see Dawson, 2008). By the latest Eocene (Chadronian NALMA), North American lagomorphs became quite abundant (e.g., Dawson, 2008), diverging into few distinct lineages, *Megalagus*, and especially *Palaeolagus*, being the most common and widespread (Fostowicz‐Frelik, 2013).

Concerning the comprehensive anatomy of the bony labyrinth in extant lagomorphs, only the inner ear structures of *Oryctolagus cuniculus* have been studied in detail (Abd El‐Hameed et al. (2023) for CT and MRI imaging; Wysocki et al. (2007) for the topographical anatomy of the temporal). Recently, the first bony labyrinth for a fossil lagomorph (*Palaeolagus haydeni*, an early Oligocene species) has been described (Ruf et al., 2021). However, *Megalagus* is a member of a more basal lineage of early lagomorphs (Fostowicz‐Frelik & Meng, 2013; see also López‐Torres et al., 2020) and the earliest lagomorph for which the structure in question is known, making it of arguably greater relevance to understanding primitive stages for the order.

In this paper, we use high‐resolution X‐ray CT data to provide the description of a digital endocast of the inner ear of the early lagomorph *Megalagus turgidus* and reconstruct the locomotor agility and hearing sensitivity of this extinct species compared with those of modern lagomorphs.

## 2 MATERIALS AND METHODS

Our study focuses on the otic region of *Megalagus turgidus*, reconstructed using CT data of an almost complete cranium (FMNH UC 1642) from the early Oligocene (early Orellan), Brule Formation of Grime's Ranch, Sioux County, Nebraska (Dawson, 1958; Olson, 1942).

**FIGURE 1:**
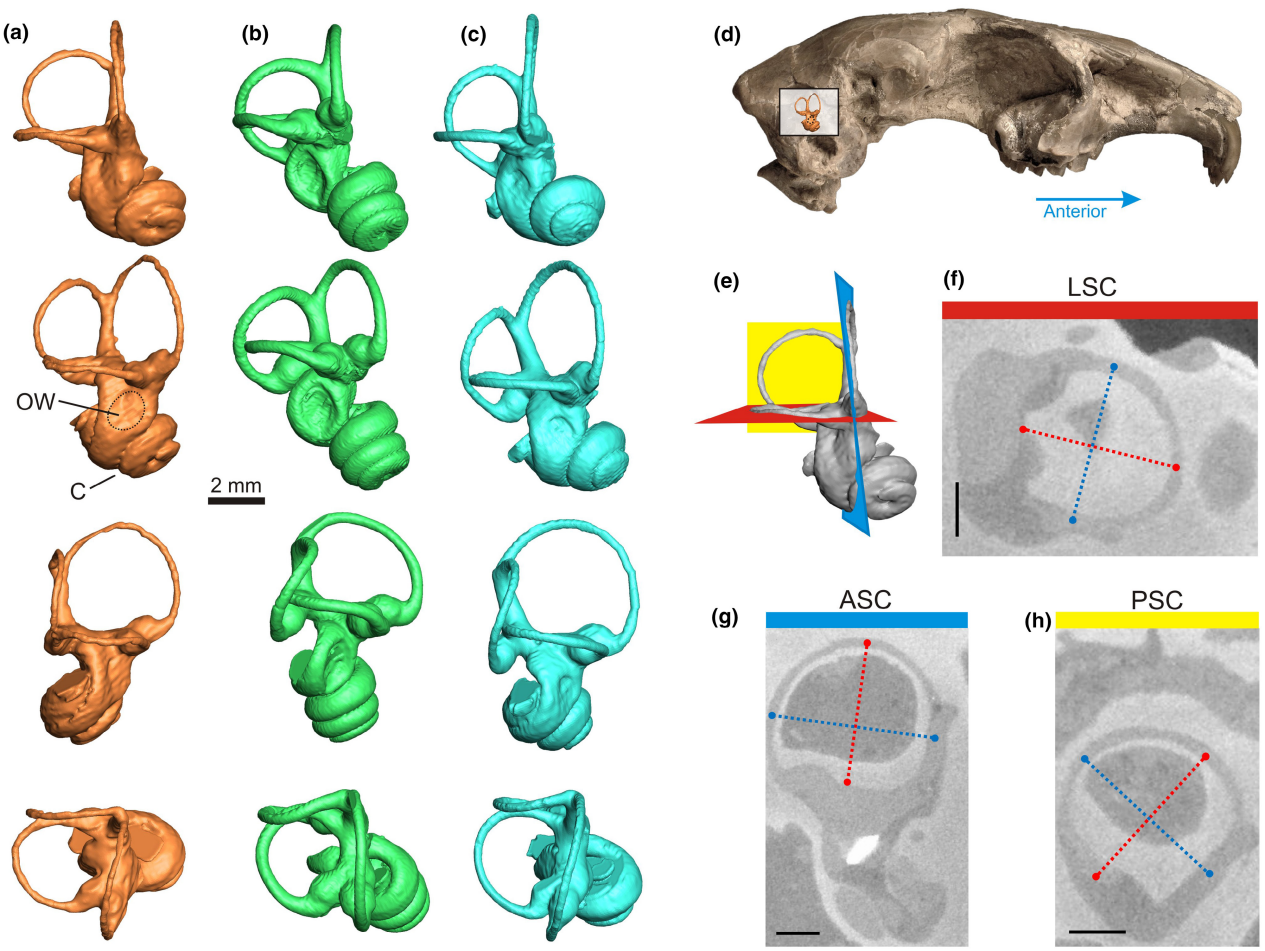
Digital visualization of the bony labyrinth in *Megalagus turgidus* (a), extant ochotonid *Ochotona pallasi* (b), and leporid *Lepus americanus* (c). Semicircular canals in each column from top to bottom in anterolateral, lateral, posterolateral, and dorsal views. Location of the bony labyrinth within the skull of *Megalagus turgidus* (d). Orientations of the semicircular canals: LSC (red), ASC (blue), and PSC (yellow) shown in (e). Measurements: height (red dotted line) and width (blue dotted line) of the semicircular canals in their respective planes are shown in f, g, h; following Spoor et al. (2007). Scale bars in (f), (g), and (h) represent 1 mm. ASC, anterior semicircular canal; C, cochlea; LSC, lateral semicircular canal; OW, oval window; PSC, posterior semicircular canal.

**FIGURE 2:**
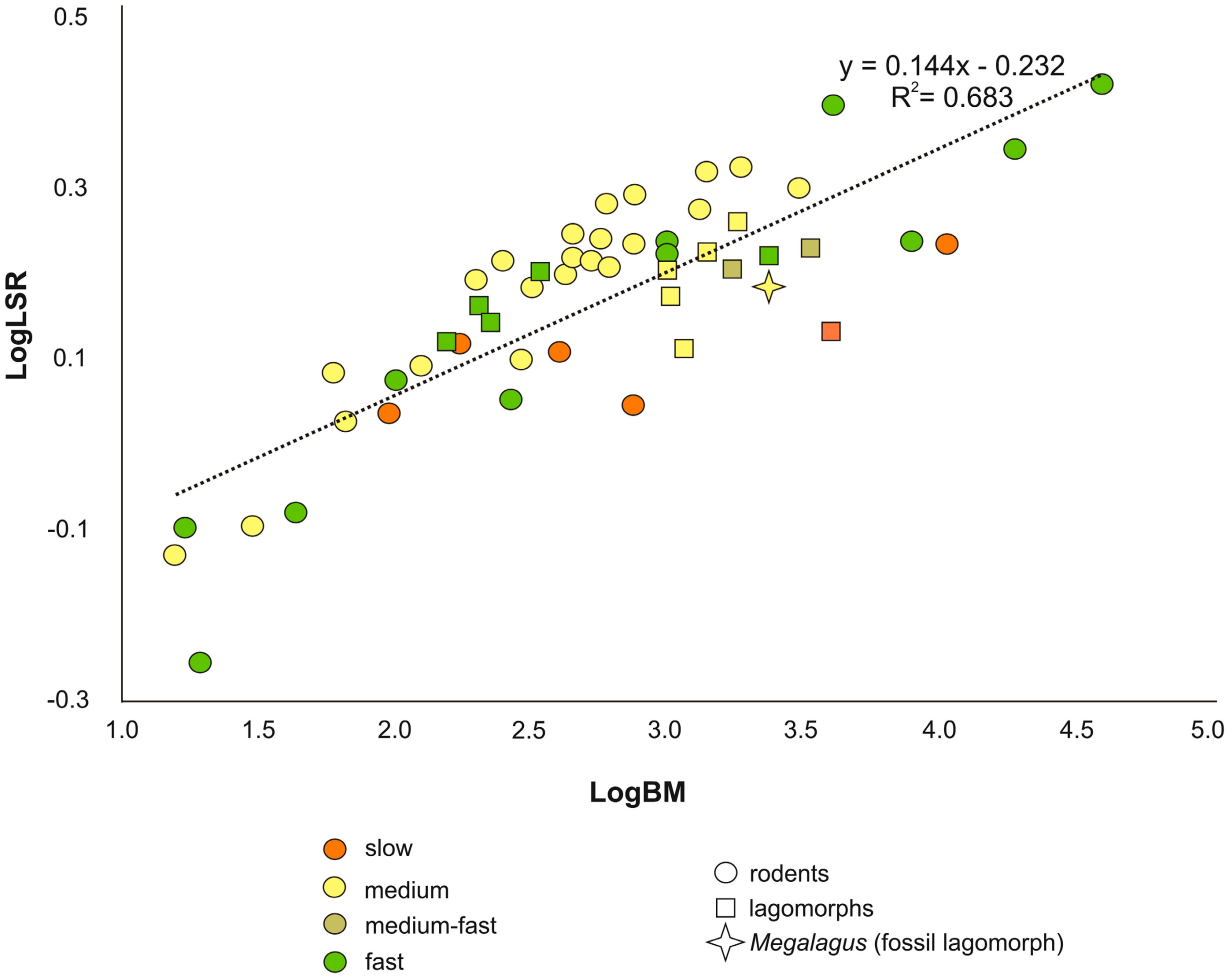
Relationship between body mass (BM) and lateral semicircular canal radius (LSR) for extant lagomorphs and rodents, and *Megalagus*. *Megalagus* marked with yellow asterisk. Linear ordinary least squares regression is based on raw data in this paper (lagomorphs; see Appendix A), Ekdale (2013; *Lepus californicus* and *Sylvilagus floridanus*), and Spoor et al. (2007; rodents). For simplification, our “medium” category designation includes categories 3 (“medium slow”) and 4 (“medium”) of Spoor et al. (2007).

**FIGURE 3:**
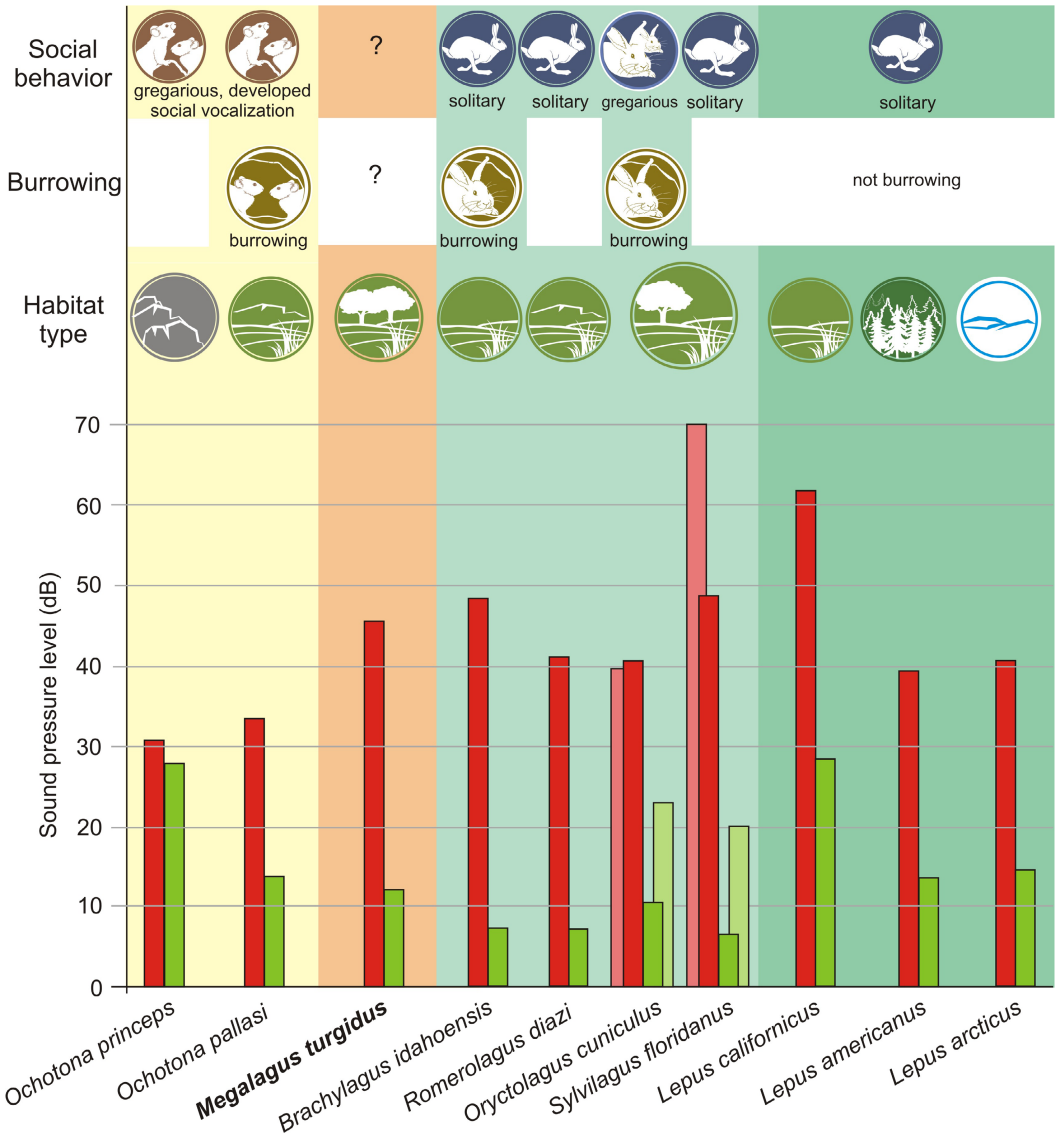
Comparisons of hearing sensitivity of *Megalagus* to extant lagomorphs. The estimations based on predicted low‐frequency and high‐frequency hearing sensitivity. The SPL at 250 Hz (SPL_250Hz_; red) was used as a proxy for low‐frequency sensitivity and sound pressure at 32 kHz (SPL_32kHz_; green) as a proxy for high‐frequency sensitivity. The lower the sound pressure is, the more increased is the sensitivity. Actual data from behavioral audiograms for *Oryctolagus cuniculus* (from Heffner & Masterton, 1980) and *Sylvilagus floridanus* (from Heffner et al., 2020) in light red (SPL_250Hz_) and light green (SPL_32kHz_), respectively. Some ecological and behavioral traits (social and burrowing behavior, and preferred landscape) marked on the chart for the particular groups: pikas (*Ochotona*; yellow), *Megalagus* (orange), rabbits (light green), and hares (*Lepus*; dark green). Qualitative data on extant lagomorphs from Smith et al. (2018).

The cranium of *Megalagus turgidus* was scanned at the X‐ray computed tomography scanner (Phoenix v|tome|x L 240 scanner; GE Measurement & Control Solutions) at the Microscopy and Imaging Facility of the American Museum of Natural History. TIFF images of the CT data were visualized in ImageJ (Schneider et al., 2012) and cropped around the bony labyrinth for each specimen using WACOM Cintiq 21UX tablet. The data were resliced using Avizo® 7.0.1 (Visualization Sciences Group, 1995‐2012) software so that each semicircular canal (SCC) could be visualized in a single plane (Figure 1; see also Spoor et al., 2007). Images of the cross sections were further analyzed and measured (height and width for each SCC) in ImageJ. We used the better preserved right inner ear endocast for the full reconstruction (Figure 1). The bony labyrinth structure of *Megalagus* was further compared with data from extant lagomorphs (leporids and ochotonids), and a variety of modern and extinct Glires (see Figures 1, 2, 3; Table 1; for raw data see Appendix A).

**TABLE 1:** Measurements and parameters of the bony labyrinth structures of *Megalagus turgidus*.

Measurement (abbreviation); values in mm	
Anterior semicircular canal height (ASH)	3.43
Anterior semicircular canal width (ASW)	3.92
Anterior semicircular canal radius (ASR)	1.84
Posterior semicircular canal height (PSH)	2.68
Posterior semicircular canal width (PSW)	3.09
Posterior semicircular canal radius (PSR)	1.44
Lateral semicircular canal height (LSH)	3.13
Lateral semicircular canal width (LSW)	2.99
Lateral semicircular canal radius (LSR)	1.53
Cochlear length (CL)	15.70
Major axis of oval window (M)	1.05
Minor axis of oval window (m)	0.66
Oval window area (OWA)	0.55
Ratio	
ASH/ASW	0.87
PSH/PSW	0.87
LSH/LSW	1.05
ASR/LSR	1.29
ASR/PSR	1.27
PSR/LSR	0.94

*Note*: For comparative data of extant Lagomorpha, see Appendix A. “Agility score” based on LSR and agility category according to Spoor et al. (2007) are 3.59 and “medium,” respectively.

We estimated the locomotor agility of *Megalagus* using an agility score, which was calculated following equations provided by Spoor et al. (2007) and Silcox et al. (2009). The latter paper presented regression equations to calculate agility scores for mammals based on each SCC radii (ASR, PSR, and LSR) as well as an equation based on the average radius for the three SCCs. According to Silcox et al. (2009), the radius of the lateral semicircular canal (LSC) is the best predictor of agility level, probably because the LSC is the least constrained by the size and morphology of the petrosal lobule (Jeffery et al., 2008). Therefore, we are calculating agility scores based on the radius of the LSC. The agility score of Spoor et al. (2007) ranges in scale from 1 to 6, with one being extremely slow and six fast animals. Although agility scores for the lagomorph specimens in our sample (see Appendix A: Table A1) are calculated considering the qualitative approach used by Spoor et al. (2007) in assigning agility categories, we also examine data directly through bivariate plots of log_10_LSR versus log_10_BM (BM, body mass) for the combined sample of our new lagomorph specimens and Spoor et al. (2007) lagomorph data (Appendix A).

Previous research on the functional morphology of the auditory system in living euarchontans (Coleman, 2007; Coleman & Colbert, 2010) found a strong linear relationship between cochlear length (CL) and sound pressure level (SPL) at 250 Hz, and a strong, but less so association between the oval window area (OWA) and SPL at 32 kHz. CL and OWA were estimated by measuring the outer circumference of the cochlear canal and the major (M) and minor (m) axes of the oval window, respectively, following Coleman and Boyer (2012). Whereas these equations generate quantitative estimates of frequency sensitivity in Euarchonta, no members of Glires were included in the original sample. Therefore, while we employ these relationships, the quantitative results should be treated as indicative. We assumed the SPL at 250 Hz as a threshold for measuring low‐frequency sensitivity and SPL at 32 kHz for high‐frequency sensitivity after Coleman and Boyer (2012). High‐ and low‐frequency thresholds are measured in decibels (dB) and indicate how sensitive an animal's hearing is relative to another. A lower threshold is indicative of more sensitivity to a particular hearing frequency compared with a higher threshold.

## 3 RESULTS

### 3.1 Structure of the bony labyrinth

The morphology of the cochlea and SCCs in *Megalagus* resembles closely that in *Palaeolagus* (see Ruf et al., 2021), differing slightly in the SCCs shape and their spatial arrangement. The cochlea of *Megalagus* is tightly coiled and conic, but relatively flat; it has two turns approximately and is a bit shorter than that of *Palaeolagus* (Ruf et al., 2021). Contrary to the condition in modern lagomorphs, the basal turn is not in full contact with the apical turns of the cochlea. The linear length of the cochlear canal in *Megalagus turgidus* is 15.70 mm and falls at the lower end of the range for modern leporids (14.46–18.72 mm; see Appendix A) and is much lower than the range for ochotonids (20.47–22.05 mm). All three SCCs in *Megalagus turgidus* are thinner than in modern lagomorphs and have well‐pronounced ampullae, although less inflated than in modern taxa (Figure 1). The canals show almost no planar deviation, apart from the slight undulation of ASC, which is a contrast between *Megalagus* and modern lagomorphs. There is some undulation of the PSC in leporids and even more pronounced undulation of the PSC and LSC in ochotonids (see Figure 1). A slightly undulating PSC is also visible in *Palaeolagus* (see Ruf et al., 2021: figure 5), although much less than in modern taxa.

The ASC in *Megalagus* has the largest radius (ASR = 1.96 mm; Table 1; see Appendix A) of the three canals, similar to *Palaeolagus* (Ruf et al., 2021), modern lagomorphs, other Glires (including *Rhombomylus*; see Meng et al., 2003), and plesiadapiforms (Silcox et al., 2009). Interestingly, the PSC of *Megalagus* has the shortest radius, in contrast to other lagomorphs (e.g., *Lepus arcticus* or *Ochotona pallasi*) as well as to *Rhombomylus*, in which the shortest radius is found for the LSC. Compared with *Palaeolagus*, the canals in *Megalagus* have a more regular (almost ideally circular) course, while in the former they are slightly compressed either laterally, anteriorly, or posteriorly.

Similar to *Palaeolagus haydeni*, *Megalagus turgidus* differs significantly from crown lagomorphs in exhibiting a secondary common crus, a structure absent in extant lagomorphs and regarded as plesiomorphic (see Ruf et al., 2021). Its presence derives from the relative position of the LSC with respect to the PSC, where the inferior end of the latter reaches as far down as the plane defined by the LSC and meets the posterior end of the LSC, causing them to have a common course for a short distance and share also the hollow space containing the posterior ampulla (Figure 1). In modern lagomorphs, the inferior end of the PSC extends much lower than the plane defined by the LSC, which goes into the vestibule separately, thereby not forming a unified secondary common crus (Ekdale, 2013; Ruf et al., 2021).

The round window (fenestra cochleae) in *Megalagus turgidus* does not extend posteriorly beyond the PSC, similar to *Palaeolagus* (Ruf et al., 2021) and modern leporids. It is posterolateral to the oval window (fenestra vestibuli) and is directed posterolaterally with a dorsal inclination. Modern ochotonids have a different arrangement of these structures with the round window positioned directly posterior to the oval window and directed dorsolaterally. The oval window of *Megalagus* is smaller and less marked than in modern *Ochotona*, resembling the condition in leporids and *Palaeolagus*.

### 3.2 Locomotor agility

Spoor et al. (2007) observed that more agile animals tend to have larger radii of the SCCs for a given body mass in a sample of 210 living mammal species including two leporids (*Lepus europaeus* and *Oryctolagus cuniculus*). They identified “agility” with speed, and the analyzed species were grouped into six agility categories. Our sample includes only the Glires from Spoor et al. (2007), which are categorized as slow (2), medium (4), and fast (6). No Glires were represented for the extremely slow (1) and medium‐slow (3) categories; the medium‐fast (4) category was made up exclusively of the two leporid species included in Spoor et al. (2007). Our more extensive lagomorph sample (including rabbits, hares, and extant pikas [*Ochotona*], as well as the extinct *Megalagus*) better captures the diversity of the group. The results show that the ochotonids, small (150–250 g) and rather slow lagomorphs, and the smallest living leporid *Brachylagus* (Smith et al., 2018) have higher inferred agility scores than the larger leporids (Figure 2). The latter group is known for their excellent cursorial abilities, especially well‐expressed in true hares (*Lepus*). Such results suggest that linear speed and maneuverability, although closely related, are quite different phenomena. Agility can be considered in terms of the frequency and erraticism of head movement (Jeffery & Cox, 2010). These are functionally related not only to fast locomotion but also to quick response to visual cues. In lagomorphs, our conclusion is supported further by behavioral clues: pikas that inhabit mostly the rocky habitat of high mountains (talus patches) or semidesert mountain foothills are constantly challenged by their environment to move swiftly among boulders, climbing unstable substrates, and squeezing through crevices. Such locomotion requires high maneuverability and quick response to surface changes. Furthermore, the pygmy rabbit (*Brachylagus idahoensis*), the only leporid showing an unexpectedly high agility score (Figure 2) is at the same time the only leporid which does not leap effectively, but rather hops quickly, zigzagging in dense sagebrush cover (Green & Flinders, 1980).

In general, most analyzed leporids as well as *Megalagus* (agility score = 3.6) fall among the Glires of medium agility, or as a slow‐moving member in the case of *Lepus arcticus* (one of the largest species among living lagomorphs). These results indicate a strong negative dependence of agility scores on body mass in Lagomorpha.

Compared with *Megalagus*, among the fossil Glires only ischyromyid rodents have similar scores to *M. turgidus* (3.2–4.4), whereas fossil sciurids (5.5–5.7) and aplodontiids (4.1–6.1) display higher agilities scores (see Bhagat et al., 2021).

### 3.3 Hearing range

The hearing sensitivity of *Megalagus* reconstructed for the low‐frequency sound (SPL at 250 Hz; 45.23 dB) falls within the range for modern leporids (37.89–62.02 dB; close to *Brachylagus* [48.26 dB]), which are generally less sensitive than modern ochotonids in this respect (29.78–33.63 dB; Figure 3; see Appendix A for details). On the contrary, SPL reconstructed at 32 kHz indicates that *Megalagus* perceived high‐frequency sounds at 12.08 dB, which makes this species more sensitive than pikas (13.83–28.58 dB) and all analyzed hares (12.77–27.16 dB) but was less sensitive than rabbits (10.72 dB; Figure 3).

Only a handful of studies exists on behaviorally tested hearing sensitivity in Lagomorpha, which allows us to compare our estimates with actual values. According to Heffner and Masterton (1980), the low‐frequency sensitivity for *Oryctolagus* is 39.5 dB, which agrees with our estimate of 40.7 dB, and the high‐frequency sensitivity is 20–26 dB versus predicted 10.7 dB (Figure 3; Table A4). The Eastern cottontail (*Sylvilagus floridanus*) exhibits low‐frequency sensitivity of 67–77 dB (Heffner et al., 2020) versus predicted 57.9 dB (Table A4), and high‐frequency sensitivity ~20 dB (Heffner et al., 2020) versus predicted 6.3 dB (Table A4). Thus, the discrepancies between experimental and estimated data are about 10 dB. This is comparable to the intraspecific range of variation for rodents, for example, *Cynomys ludovicianus* (measured low‐frequency sensitivity 25–36.5 dB, high‐frequency sensitivity 75– over 92 dB; Heffner et al., 1994), or guinea pig (measured low‐frequency sensitivity 25–46 dB, high‐frequency sensitivity 5–16 dB; Heffner et al., 1971).

## 4 DISCUSSION

The agility score and dimensions of the inner ear structures (see Appendix A for details) of *Megalagus turgidus* are more similar to those of extant leporids such as *Lepus americanus* or *L*. *arcticus*. These two last species are not noted for their great agility. They inhabit boreal regions of North America, covered by taiga or tundra type of vegetation, and having a deep snow cover during the winter. These factors hamper both fast locomotion and maneuverability, although the Arctic hare is known for its endurance to cover long distances (Lai et al., 2022). In the case of *Megalagus*, the reconstructed environment of the early Oligocene Brule Formation indicates open woodland habitat with abundant bushes and restricted grassy and herbaceous openings (Hutchinson, 1989; Leopold et al., 1992; Retallack, 1983), which would have been similar to the habitat of extant *Lepus americanus*. Dawson (1958) considered the postcranial evidence in *Megalagus turgidus* and concluded that this species was not a rapid cursor and may have had a similar locomotor behavior to that of present‐day pikas (*Ochotona*).

With respect to the SCC proportions, Schmelzle et al. (2007) observed that in marsupial species which stand in a more erect posture, like kangaroos and wallabies (genus *Macropus*), the ASC is much taller than the PSC in comparison with species with a rather horizontal posture, in which the ASC and the PSC had a similar height. Interestingly, all lagomorphs also have the ASC generally taller than the PSC, especially leporids. However, *Megalagus* has a similar ASC‐to‐PSC ratio to ochotonids, which display a more uniform height between the anterior and posterior SCCs. Leporids do not routinely exhibit an erect posture, contrasting in this way from *Macropus*, for example, but they do share to certain extent a saltatorial (although not ricochetal) type of locomotion with kangaroos and wallabies. On the contrary, ochotonids and *Megalagus* do not share the typical leaping‐gallop locomotion of leporids, having a rather ambulatory locomotion. Also, a higher ASC with respect to the PSC is observed in *Leptictidium*, a saltatorial leptictidan, compared with *Leptictis* and *Palaeoictops*, nonsaltatorial leptictidans (Ruf et al., 2016). Therefore, a proportionally taller ASC with respect to the PSC may be associated with saltatorial locomotion, that is, fast and repetitive movements of the head (and body) along the vertical axis.

The estimated hearing sensitivity supports lagomorphs as better adapted to high‐frequency sounds, because all lagomorph species including *Megalagus turgidus* show lower SPLs for the high frequencies than for the low ones (Figure 3). However, our results do not fully confirm previous observations that smaller mammals have heightened high‐frequency sensitivity, which was inferred to have been mainly to take advantage of spectral cues that aid in the ability to localize the source of sound (Heffner et al., 2020; Heffner & Heffner, 2010). Ochotonids are smaller than leporids, but they are not among the species with greater hearing sensitivity to high frequencies. *Ochotona princeps* is the least sensitive to high‐frequency sounds of our entire lagomorph sample (Figure 3). This reversed pattern might be explained by the fact that ochotonids have a more complex vocalization repertoire than leporids, perform calls with a wide frequency range (Konishi, 1970; Trefry & Hik, 2010) and, in some populations, even produce multiple‐note calls (Conner, 1982). Moreover, high‐frequency calls in Altai pikas (*Ochotona alpina*) are within the range of 7.31–15.46 kHz (Volodin et al., 2018), which is probably similar to that of other pikas, and much less than 32 kHz used for high‐frequency sensitivity estimation. Although there is an overlap between leporids and ochotonids in their high‐frequency hearing sensitivity, ochotonids display higher low‐frequency sensitivity than leporids, which can be related to a more open‐landscape habitat of the former, where the low‐frequency sounds propagate easier and for longer distances.

Our results show that early lagomorphs including *Megalagus* were more leporid‐like in terms of hearing sensitivity, and accordingly, most likely did not exhibit a complex vocal repertoire typical of pikas. That also may suggest the solitary way of living for these lagomorphs similar to most of living leporids. Whereas there is still a lot that we do not know about the sensory evolution in lagomorphs, the study of the inner ear endocast of *Megalagus* is the first to help understand the leporid‐like nature of the primitive lagomorph characters associated with hearing, as well as important aspects of how early lagomorphs perceived and interacted with their environment.

## AUTHOR CONTRIBUTIONS

**Sergi López‐Torres:** Investigation (equal); validation (equal); visualization (equal); writing – original draft (lead). **Raj Bhagat:** Investigation (equal); validation (equal); writing – review and editing (equal). **Ornella C. Bertrand:** Investigation (supporting); writing – review and editing (equal). **Mary T. Silcox:** Investigation (equal); methodology (lead); resources (equal); writing – review and editing (equal). **Lucja Fostowicz‐Frelik:** Conceptualization (lead); funding acquisition (lead); supervision (lead); visualization (equal); writing – review and editing (lead).

## FUNDING INFORMATION

This research was funded by National Science Centre (Cracow, Poland) grant no. 2015/18/E/NZ8/00637 and an AMNH Roosevelt Research Fellowship to Ł.F.F, and an NSERC Discovery Grant to M.T.S.

## CONFLICT OF INTEREST STATEMENT

We declare no conflict of interest.

## Data Availability

All morphometric data that originated as a result of this study are available in Appendix A.

## APPENDIX A

See Tables A1, A2, A3, A4, A5, A6, A7.

**TABLE A1:** Comparative material and scan parameters of the lagomorph specimens used for the study.

Species	Coll. number	Scanning facility	Source‐object distance (mm)	Energy settings		Number of views	Voxel size (mm)	Columns × rows (total)	Total number of slices
				kv	mA				
*Brachylagus idahoensis*	AMNH 92869	SMIF	118.64	138	87	2000	0.045899	1331 × 1393	826
*Lepus americanus phaeonotus*	AMNH 97648	SMIF	165.24	138	87	2000	0.041922	1416 × 1413	988
*Lepus americanus bairdii*	AMNH 99352	AMNH	239.11	135	180	2250	0.041191	1114 × 1111	926
*Lepus arcticus*	AMNH 42139	SMIF	233.98	148	90	2000	0.055091	1456 × 1384	911
*Oryctolagus cuniculus*	AMNH 34816	SMIF	158.68	138	87	2000	0.040904	1255 × 1394	953.5
*Romerolagus diazi*	AMNH 48172	AMNH	91.94	150	180		0.022608	1669 × 1361	1364
*Ochotona princeps*	AMNH 120698	SMIF	87.95	129	94	2000	0.023833	1255 × 1853	897
*Ochotona princeps schisticeps*	AMNH 40547	AMNH	134.26	105	165	2350	0.023128	1583 × 1570	998
*Ochotona pallasi*	AMNH 59712	SMIF	89.27	123	98	2000	0.024188	1033 × 1077	906
*Megalagus turgidus*	FMNH UC 1642	AMNH	91.68	155	145	2250	0.022544	1564 × 1421	2701

**TABLE A2:** Metrical data of the width of the occipital condyles and BM estimation (using formula of Moncunill‐Solé et al., 2015) for studied lagomorph taxa.

Species	Specimen	Occipital W (in mm)	Body mass (in g)
*Brachylagus idahoensis*	AMNH 92869	9.81	339.5
*Lepus americanus phaeonotus*	AMNH 97648	12.77	998.6
*Lepus americanus bairdii*	AMNH 99352	13.86	1396.2
*Lepus arcticus*	AMNH 42139	17.93	4003.1
***Megalagus turgidus***	FMNH UC 1642	15.70	2325.0
*Ochotona pallasi*	AMNH 59712	8.86	223.8
*Ochotona princeps princeps*	AMNH 120698	8.65	202.9
*Ochotona princeps schisticeps*	AMNH 40547	8.10	155.1
*Oryctolagus cuniculus*	AMNH 34816	14.74	1796.1
*Romerolagus diazi*	AMNH 48172	12.86	1027.8

**TABLE A3:** Agility scores and categories for *Megalagus turgidus* and living lagomorphs, assigned following Spoor et al. (2007).

Species	Specimen	Agility score from LSR	Agility category
*Megalagus turgidus*	FMNH UC 1642	3.59	Medium
*Brachylagus idahoensis*	AMNH 92869	5.07	Fast
*Lepus americanus bairdii*	AMNH 99352	4.22	Medium
*Lepus americanus phaeonotus*	AMNH 97648	4.28	Medium
*Lepus arcticus*	AMNH 42139	2.94	Slow
*Oryctolagus cuniculus*	AMNH 34816	4.33	Medium
*Romerolagus diazi*	AMNH 148172	4.02	Medium
*Ochotona pallasi*	AMNH 59712	4.88	Fast
*Ochotona princeps*	AMNH 120698	5.15	Fast
*Ochotona princeps schisticeps*	AMNH 40547	4.94	Fast

*Note*: LSR = radius of the lateral semicircular canal.

**TABLE A4:** Cochlear measurements for *Megalagus turgidus* and living lagomorphs (in mm) and predicted low‐frequency and high‐frequency hearing sensitivity.

Species	Specimen	CL	*M*	*m*	OWA	Low frequency (250 Hz)	High frequency (32 kHz)
*Megalagus turgidus*	FMNH UC 1642	15.70	1.05	0.66	0.55	45.23	12.08
*Brachylagus idahoensis*	AMNH 92869	14.46	0.80	0.52	0.33	48.26	7.35
*Lepus americanus bairdii*	AMNH 99352	18.72	1.20	0.75	0.70	37.89	15.35
*Lepus americanus phaeonotus*	AMNH 97648	17.35	1.00	0.74	0.58	41.22	12.77
*Lepus arcticus*	AMNH 42139	17.58	1.16	0.73	0.67	40.65	14.62
*Lepus californicus*	TMM M‐7500	8.80	1.64	0.97	1.25	62.02	27.16
*Oryctolagus cuniculus*	AMNH 34816	17.57	1.04	0.60	0.49	40.68	10.72
*Romerolagus diazi*	AMNH 148172	17.45	0.72	0.56	0.32	40.98	7.09
*Sylvilagus floridanus*	TMM M‐5987	10.5	0.96	0.37	0.28	57.88	6.26
*Ochotona princeps princeps*	AMNH 120698	21.20	1.52	1.05	0.63	33.63	13.83
*Ochotona princeps schisticeps*	AMNH 40547	22.05	1.60	1.05	1.25	31.84	27.23
*Ochotona pallasi*	AMNH 59712	20.47	1.05	0.77	1.32	29.78	28.58

Abbreviations: CL, cochlear length; M = major axis of the oval window; m = minor axis of the oval window; OWA, oval window area.

**TABLE A5:** Semicircular canal measurements for *Megalagus turgidus* and living lagomorphs (in mm).

Species	Specimen	ASC		PSC		LSC	
		*h*	*w*	*h*	*w*	*h*	*w*
*Megalagus turgidus*	FMNH UC 1642	3.43	3.92	2.68	3.09	3.13	2.99
*Brachylagus idahoensis*	AMNH 92869	4.38	5.75	1.89	2.74	3.18	3.13
*Lepus americanus bairdii*	AMNH 99352	4.21	4.77	3.02	3.23	3.32	3.39
*Lepus americanus phaeonotus*	AMNH 97648	4.21	4.84	2.62	3.47	3.00	3.39
*Lepus arcticus*	AMNH 42139	4.38	5.1	2.88	3.05	2.13	3.27
*Oryctolagus cuniculus*	AMNH 34816	4.25	4.93	3.04	3.21	3.61	3.65
*Romerolagus diazi*	AMNH 148172	3.56	4.34	2.59	2.64	2.90	3.06
*Ochotona princeps princeps*	AMNH 120698	2.44	3.68	1.98	2.97	2.72	3.08
*Ochotona princeps schisticeps*	AMNH 40547	2.76	3.18	2.55	2.76	2.42	2.83
*Ochotona pallasi*	AMNH 59712	2.56	3.49	2.5	2.75	2.72	2.83

Abbreviations: ASC, anterior semicircular canal; LSC, lateral semicircular canal; h, height; PSC, posterior semicircular canal; w, width.

**TABLE A6:** Radii of the semicircular canals of *Megalagus turgidus* and living lagomorphs (in mm), and semicircular canal proportions.

Species	Specimen	ASR	PSR	LSR	SCR	ASC h/w	PSC h/w	LSC h/w
*Megalagus turgidus*	FMNH UC 1642	1.84	1.44	1.53	1.60	0.87	0.87	1.05
*Brachylagus idahoensis*	AMNH 92869	2.53	1.16	1.58	1.76	0.76	0.69	1.02
*Lepus americanus bairdii*	AMNH 99352	2.25	1.56	1.68	1.83	0.88	0.93	0.98
*Lepus americanus phaeonotus*	AMNH 97648	2.26	1.52	1.60	1.79	0.87	0.76	0.88
*Lepus arcticus*	AMNH 42139	2.37	1.48	1.35	1.73	0.86	0.94	0.65
*Lepus californicus*	TMM M‐7500	2.34	1.69	1.66	1.90	–	–	–
*Lepus europaeus*	See Spoor et al. (2007)	2.31	1.75	1.69	1.92	–	–	–
*Oryctolagus cuniculus*	See Spoor et al. (2007)	2.00	1.69	1.59	1.76	–	–	–
*Oryctolagus cuniculus*	AMNH 34816	2.30	1.56	1.82	1.89	0.86	0.95	0.99
*Romerolagus diazi*	AMNH 148172	1.98	1.31	1.49	1.59	0.82	0.98	0.95
*Sylvilagus floridanus*	TMM M‐5987	1.86	1.44	1.29	1.53	–	–	–
*Ochotona pallasi*	AMNH 59712	2.37	1.48	1.35	1.73	0.73	0.91	0.96
*Ochotona princeps princeps*	AMNH 120698	1.53	1.24	1.45	1.05	0.66	0.67	0.88
*Ochotona princeps schisticeps*	AMNH 40547	1.49	1.33	1.31	1.38	0.87	0.92	0.86

Abbreviations: ASC, anterior semicircular canal; ASR, radius of the anterior semicircular canal; h, height; LSC, lateral semicircular canal; LSR, radius of the lateral semicircular canal; PSC, posterior semicircular canal; PSR, radius of the posterior semicircular canal; SCR, average radius of all semicircular canals; w, width. Heights and widths are in Table A4. The height/width proportions with no numerical values are those not reported by Spoor et al. (2007) and Ekdale (2013).

**TABLE A7:** Semicircular canal radius proportions for *Megalagus turgidus* and living lagomorphs.

Species	Specimen	ASR/LSR	ASR/PSR	PSR/LSR
*Megalagus turgidus*	FMNH UC 1642	1.20	1.27	0.94
*Brachylagus idahoensis*	AMNH 92869	1.61	2.19	0.73
*Lepus americanus bairdii*	AMNH 99352	1.34	1.44	0.93
*Lepus americanus phaeonotus*	AMNH 97648	1.42	1.49	0.95
*Lepus arcticus*	AMNH 42139	1.76	1.60	1.10
*Lepus californicus*	TMM M‐7500	1.41	1.38	1.02
*Lepus europaeus*	See Spoor et al. (2007)	1.37	1.32	1.04
*Oryctolagus cuniculus*	See Spoor et al. (2007)	1.26	1.18	1.06
*Oryctolagus cuniculus*	AMNH 34816	1.26	1.47	0.86
*Romerolagus diazi*	AMNH 148172	1.33	1.51	0.88
*Sylvilagus floridanus*	TMM M‐5987	1.44	1.29	1.12
*Ochotona pallasi*	AMNH 59712	1.09	1.15	0.95
*Ochotona princeps princeps*	AMNH 120698	1.06	1.24	0.85
*Ochotona princeps schisticeps*	AMNH 40547	1.13	1.12	1.01

Abbreviations: ASR = radius of the anterior semicircular canal; LSR = radius of the lateral semicircular canal; PSR = radius of the posterior semicircular canal.
